# Unraveling the Metabolic Requirements of the Gut Commensal *Bacteroides ovatus*

**DOI:** 10.3389/fmicb.2021.745469

**Published:** 2021-11-25

**Authors:** Robert Fultz, Taylor Ticer, Faith D. Ihekweazu, Thomas D. Horvath, Sigmund J. Haidacher, Kathleen M. Hoch, Meghna Bajaj, Jennifer K. Spinler, Anthony M. Haag, Shelly A. Buffington, Melinda A. Engevik

**Affiliations:** ^1^Department of Neuroscience, Cell Biology, and Anatomy, University of Texas Medical Branch, Galveston, TX, United States; ^2^Department of Regenerative Medicine & Cell Biology, Medical University of South Carolina, Charleston, SC, United States; ^3^Department of Pediatrics, Baylor College of Medicine, Houston, TX, United States; ^4^Section of Gastroenterology, Hepatology, and Nutrition, Texas Children’s Hospital, Houston, TX, United States; ^5^Department of Pathology and Immunology, Baylor College of Medicine, Houston, TX, United States; ^6^Department of Pathology, Texas Children’s Hospital, Houston, TX, United States; ^7^Department of Chemistry and Physics and Department of Biotechnology, Alcorn State University, Lorman, MS, United States

**Keywords:** Bacteroides, metabolism, carbohydrates, polysaccharides, intestine, commensal

## Abstract

**Background:** Bacteroidetes are the most common bacterial phylum in the mammalian intestine and the effects of several *Bacteroides* spp. on multiple facets of host physiology have been previously described. Of the *Bacteroides* spp., *Bacteroides ovatus* has recently garnered attention due to its beneficial effects in the context of intestinal inflammation. In this study, we aimed to examine model host intestinal physiological conditions and dietary modifications to characterize their effects on *B. ovatus* growth.

**Methods and Results:** Using Biolog phenotypic microarrays, we evaluated 62 primary carbon sources and determined that *B. ovatus* ATCC 8384 can use the following carbohydrates as primary carbon sources: 10 disaccharides, 4 trisaccharides, 4 polysaccharides, 4 polymers, 3 L-linked sugars, 6 D-linked sugars, 5 amino-sugars, 6 alcohol sugars, and 15 organic acids. Proteomic profiling of *B. ovatus* bacteria revealed that a significant portion of the *B. ovatus* proteome contains proteins important for metabolism. Among the proteins, we found glycosyl hydrolase (GH) familes GH2, GH5, GH20, GH 43, GH88, GH92, and GH95. We also identified multiple proteins with antioxidant properties and reasoned that these proteins may support *B. ovatus* growth in the GI tract. Upon further testing, we showed that *B. ovatus* grew robustly in various pH, osmolarity, bile, ethanol, and H_2_O_2_ concentrations; indicating that *B. ovatus* is a well-adapted gut microbe.

**Conclusion:** Taken together, we have demonstrated that key host and diet-derived changes in the intestinal environment influence *B. ovatus* growth. These data provide the framework for future work toward understanding how diet and lifestyle interventions may promote a beneficial environment for *B. ovatus* growth.

## Introduction

The order Bacteroidales, in the phylum Bacteroidetes, is the most abundant gram-negative bacteria in the human gut (Rios-Covian et al., 2017), with an estimated density of 5–8 × 10^10^ CFU per gram of feces (Zitomersky et al., 2011). Bacteroides and Prevotella dominate the Bacteroidales order and Bacteroides, in particular, has become a major focus as a gastrointestinal commensal microbe (Wexler and Goodman, 2017). Bacteroides colonize the gastrointestinal tracts of mammals, including humans, and can establish stable, long-term synergistic relationships with hosts, thereby conferring numerous health benefits (Mazmanian et al., 2005; Hudcovic et al., 2009; Round and Mazmanian, 2010; Faith et al., 2013; Chiu et al., 2014; Wexler and Goodman, 2017; Ihekweazu et al., 2019, 2021; Tan et al., 2019). Since Bacteroides are major constituents of the human gut microbiota and form a foundational part of the gastrointestinal microbial food web, *Bacteroides* spp. are considered an ideal model group for investigating the fundamental principles of microbial colonization (Wexler and Goodman, 2017). One mechanism of colonization implemented by *Bacteroides* spp. is the ability to utilize a wide range of dietary polysaccharides and intestinal mucins (Sonnenburg et al., 2005; Martens et al., 2011). Bacteroides harbor multiple glycosyl hydrolases required to degrade dietary and host-derived glycans, as well as the ability to use amino acids (Smith and Macfarlane, 1998). For example, *B. thetaiotaomicron* can metabolize more than a dozen plant- and host-derived polysaccharides (Salyers et al., 1977; McNeil, 1984). To date, the majority of Bacteroides work has focused on *B. thetaiotaomicron*, while fewer studies have examined the dietary utilization of other *Bacteroides* spp. like *B. fragilis, B. vulgatus*, or *B. ovatus*.

Human-derived *B. ovatus* is a candidate next-generation probiotic because specific strains can suppress inflammation in the GI tract (Hudcovic et al., 2009; Ihekweazu et al., 2019, 2021; Tan et al., 2019). We have previously demonstrated that *B. ovatus* ameliorates colitis in dextran sodium sulfate (DSS) (Ihekweazu et al., 2019) and trinitrobenzene sulfonic acid (TNBS) (Ihekweazu et al., 2021) mouse models. We need to better understand the dietary and host-derived compounds that promote *B. ovatus’* maintenance in the intestine in order to translate the beneficial health effect observed with *B. ovatus* in model systems to patients. In this study, we examined which carbohydrates, polysaccharides, organic nitrogen sources, and other compounds could affect the growth of *B. ovatus* using microbial phenotype microarray technology, genome analysis, and proteomics. This work is among the first to delineate the metabolic profile of *B. ovatus* ATCC 8384. Our data suggest that *B. ovatus* utilizes a wide range of physiologically abundant dietary nutrient sources.

## Materials and Methods

### Bacterial Culture Conditions

*Bacteroides ovatus* ATCC 8384 (ATCC, American Type Culture Collection) was grown in Brain-Heart-Infusion media (Difco) supplemented with 2% yeast extract and 0.2% cysteine in an anaerobic workstation (Anaerobe Systems AS-580) in a mixture of 5% CO_2_, 5% H_2_, and 90% N_2_ at 37°C overnight. Bacterial growth was measured by optical density (OD_600__nm_) using a spectrophotometer and adjusted to an optical density (OD_600__nm_) = 0.01 in a chemically defined minimal media (CDMM) (Karasawa et al., 1995), devoid of glucose. Then 100 μL of the *B. ovatus* CDMM solution was added to each well of 96-well Biolog microarray plates (PM1, PM2, PM5, PM9, and PM10), which were promptly sealed. Growth was monitored by OD_600__nm_ readings at 15 min intervals for 24 h on a Cerillo plate reader incubated anaerobically at 37°C. Growth was assessed compared to a negative control well lacking any carbon substrate (A1 of each plate) and a value of OD_600__nm_ ≥ 0.2 was considered as growth (*n* = 2 independent biological replicates).

To mirror the gastrointestinal tract, CDMM containing glucose was adjusted to pH 7, 6, 5, and 4 using 5 M HCl. To create a range of osmolarities, 0.1, 0.5, or 1 M NaCl (Sigma # S9888) was added to CDMM containing glucose. Likewise, stressors bovine bile (Sigma #B3883), H_2_0_2_ (Sigma # HX0636), and 200-proof ethanol (Sigma # EX0276) were individually added to CDMM medium solutions that each contained glucose. Overnight cultures of *B. ovatus* were adjusted to an OD_600nm_ = 0.01 in the CDMM containing glucose and various stressors and a 100 μL volume of the *B. ovatus* CDMM solution was added to each well of a 96-well plate (VWR #1081-562). Growth was monitored after 24 h using the plate reader.

### Proteomic Analysis

#### Chemicals and Chromatography

Proteomic analysis of *B. ovatus* ATCC 8384 was performed as previously described (Engevik et al., 2021). For all experiments, optima LC/MS-grade acetonitrile (ACN), formic acid (FA), and Promega™ porcine trypsin protease were all purchased from Thermo Fisher Scientific, while ammonium bicarbonate (BioUltra-grade) was purchased from Millipore-Sigma. Briefly, *B. ovatus* was grown anaerobically overnight in CDMM, and cultures were centrifuged at 7,000 × *g* for 5 min. *B. ovatus* pellets were suspended in a 200-μL volume of water and samples were sonicated in an ultrasonic bath for 30 min. The disrupted bacteria were then centrifuged at 10,000 × *g* for 5 min. The resulting cell-free supernatants containing bacterial proteins were dried in a SpeedVac overnight. A 100-μL volume of a 10 μg/mL solution of porcine trypsin in 25 mM ammonium bicarbonate was added to the dried proteins and incubated at 37°C for 8 h. Digested samples were chromatographically separated on a Dionex Ultimate 3000 RSLC nano-system (Thermo Scientific) using an Acclaim PepmapTM C-18 capillary column [75 μm (ID) × 150 mm (L), Thermo Scientific] outfitted with an Acclaim PepmapTM C18 trap column [100 μm (ID) × 20 mm (L), Thermo Scientific]. Chromatography was performed as previously described (Engevik et al., 2020, 2021).

#### Mass Spectrometric Analysis

Samples were analyzed as previously described (Engevik et al., 2021) using an Orbitrap Fusion mass spectrometer (Thermo Scientific) with a nanoionization source operated in positive ion mode using source and global mass spectrometer settings are previously described (Engevik et al., 2021). Resulting data were compared to the Uniprot Bacteroides database (8 Aug 2020) and analyzed using Proteome Discoverer (Thermo Scientific).

### Statistics and Graphs

All graphs were generated using GraphPad Prism software (version 9) (GraphPad Inc.). Statistical analysis was performed with Repeated Measures ANOVA with the Holm–Sidak *post-hoc* test. The data are presented as mean ± standard deviation, with *P* < 0.05 (*) considered statistically significant. See Supplementary Table 1 for statistical analysis.

## Results

**FIGURE 1:**
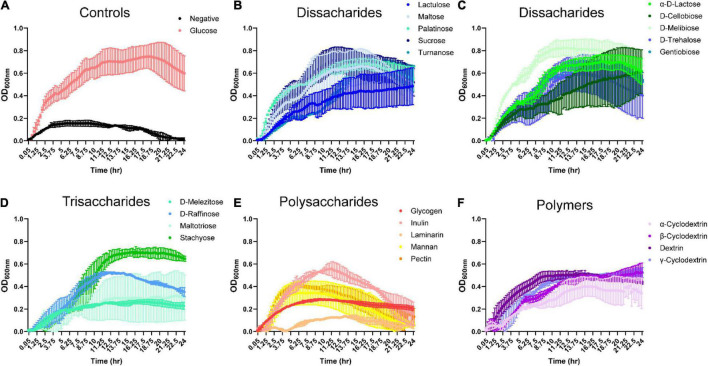
Bacteroides grows on select dissacharides, trisacharides, polysaccharides and polymers in the absence of glucose. *B. ovatus* ATCC 8384 was grown anaerobically at 37°C in Biolog plates with a chemically-defined minimal media (CDMM) preparation that lacked glucose. Growth was monitored over 24 h by plate reader in plates containing **(A)** D-glucose and no glucose controls, **(B,C)** dissaccharides, **(D)** trisaccharides, **(E)** polysaccharides, and **(F)** polymers. All data are presented as mean ± stdev.

To identify dietary compounds capable of influencing *B. ovatus* ATCC 8384 growth, we used Biolog phenotypic microarrays and a CDMM prepared without glucose. *B. ovatus* exhibited minimal growth in CDMM lacking glucose, reaching a maximal optical density (OD_600__nm_) of 0.16 ± 0.04 at 3.75 h (Figure 1A and Supplementary Figure 1A). In contrast, *B. ovatus* had robust growth in the presence of D-glucose, reaching a maximal OD_600__nm_ of 0.75 ± 0.15 at 18.25 h. Disaccharides, trisaccharides, polysaccharides and polymers are typical dietary components in the intestine that can be enzymatically digested and taken up by the gut microbiota. We monitored growth with 10 different disaccharides to determine if *B. ovatus* was capable of utilizing disaccharides for growth (Figures 1B,C). *B. ovatus* grew to an OD_600__nm_ > 0.2 (indicative of growth) with all disaccharides (lactulose, maltose, palatinose, sucrose, turnanose, α-D-lactose, D-cellobiose, D-melibiose, D-trehalose, and gentobiose; see stats in Supplementary Table 1). The greatest growth was observed with D-melibiose (OD_600__nm_ 0.83 ± 0.09 at 11.75 h), sucrose (OD_600__nm_ 0.72 ± 0.15 at 12.75 h), and maltose (OD_600__nm_ 0.66 ± 0.22 at 16 h). *B. ovatus* was also able to use the trisaccharides D-raffinose and stachyose to support growth (Figure 1D). *B. ovatus* was still able to grow to an OD_600__nm_ > 0.2 with D-melezitose and maltotriose, but the growth was far less than that observed with D-raffinose and stachyose. Of the polysaccharides, *B. ovatus* could use glycogen, inulin, mannan, and pectin, but was unable to use laminarin (Figure 1E). *B. ovatus* also utilized the polymers α-, β-, and γ-cyclodextrin as well as dextrin for growth (Figure 1F). As expected, we found that *B. ovatus* used disaccharides and trisaccharides to support the greatest growth, consistent with the notion that polysaccharides and polymers require more enzymes and time for degradation.

**FIGURE 2:**
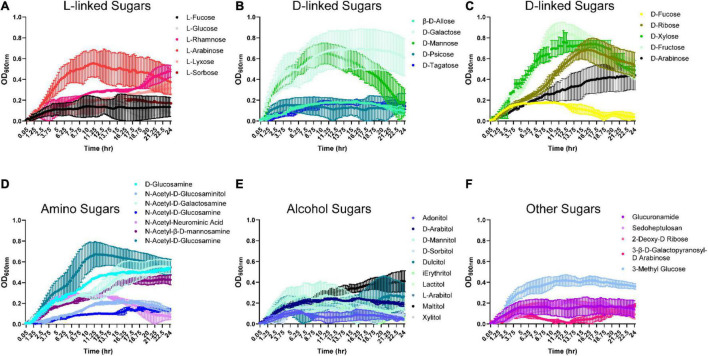
*Bacteroides ovatus* grows on certain L-, D-, amino, and alcohol modified sugars. *B. ovatus* ATCC 8384 was grown anaerobically at 37°C in Biolog plates with a chemically-defined minimal media (CDMM) preparation that lacked glucose. Growth was monitored over 24 hrs by plate reader in plates containing **(A)** L-linked sugars, **(B,C)** D-linked sugars, **(D)** amino sugars, **(E)** alcohol sugars, and **(F)** other sugars. All data are presented as mean ± stdev.

Since *B. ovatus* was efficient at utilizing disaccharides and trisaccharides for growth, we next examined the ability of *B. ovatus* to use monosaccharides. Interestingly, *B. ovatus* exhibited limited growth with L-linked sugars. *B. ovatus* was able to use three L-linked sugars: L-arabinose, L-rhamnose, and L-lyxose for growth (Figure 2A and Supplementary Figure 1B). In contrast, *B. ovatus* grew with multiple D-linked sugars, including D-ribose, D-xylose, D-fructose, D-arabinose, D-galactose, and D-mannose (Figures 2B,C). Of the D-linked sugars, *B. ovatu*s used D-fructose (OD_600__nm_ 0.85 ± 0.14 at 11.5 h) and D-galactose (OD_600__nm_ 0.70 ± 0.26 at 15 h) to generate the greatest bacterial growth. In terms of amino-sugars, *B. ovatus* utilized D-glucosamine, *N*-acetyl-D-glucosaminitol, *N*-acetyl-D-galactosamine, *N*-acetyl-neuraminic acid, and *N*-acetyl-D-glucosamine to drive replication (Figure 2D). *B. ovatus* also consumed alcohol sugars like maltitol and to a lesser degree D-arabitol, D-mannitol, lactitol, L-arabitol, and xylitol for growth (Figure 2E). When we investigated the ability of *B. ovatus* to use other sugars that occur in the gut, we observed that 3-methyl-glucose supported growth, while other compounds such as glucuronamide, sedoheptulosan, 2-deoxy-D-ribose, and 3-β-D-galactopyranosyl-D-arabinose were unable to aid *B. ovatus’* expansion (Figure 2F). These data indicate that *B. ovatus* utilizes multiple monosaccharides, disaccharides, trisaccharides, polysaccharides, and polymers to support its growth in the intestinal milieu.

**FIGURE 3:**
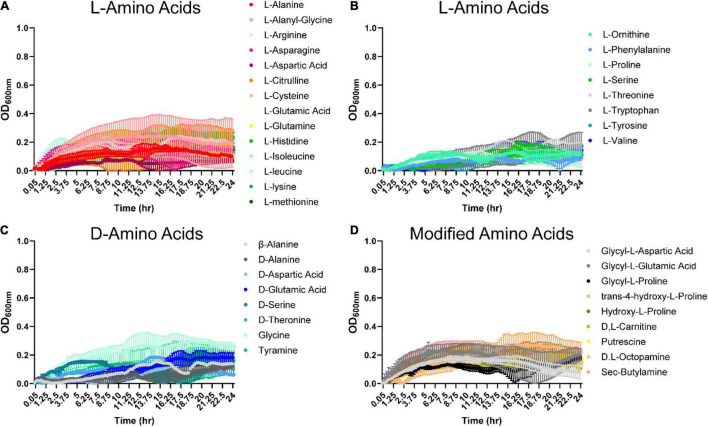
*Bacteroides ovatus* has nominal growth on amino acids in the absence of glucose. *B. ovatus* ATCC 8384 was grown anaerobically at 37°C in Biolog plates with a chemically-defined minimal media (CDMM) preparation that lacked glucose. Growth was monitored over 24 h by plate reader in plates containing **(A,B)** L-linked amino acids, **(C)** D-linked amino acids, and **(D)** modified amino acids. All data are presented as mean ± stdev.

In the gastrointestinal tract, proteins can be hydrolyzed into peptides and amino acids by bacterial- and host-derived proteases and peptidases (Macfarlane et al., 1988, 1989; Neis et al., 2015). The released amino acids can then be used by gut microbes. We were interested in identifying amino acids that could support *B. ovatus* ATCC 8384 growth in the absence of a carbon source (D-glucose). Examination of growth with L- and D-amino acids revealed that *B. ovatus* could use L-arginine, L-citrulline, L-leucine, L-lysine (Figure 3A and Supplementary Figure 1C), glycine (Figure 3C), hydroxy-L-proline, D,L-octopamine, and sec-butylamine (Figure 3D) to reach an OD_600__nm_ > 0.2, but robust growth (OD_600__nm_ > 0.5) was not observed for any of the amino acids (Figures 3A–D).

**FIGURE 4:**
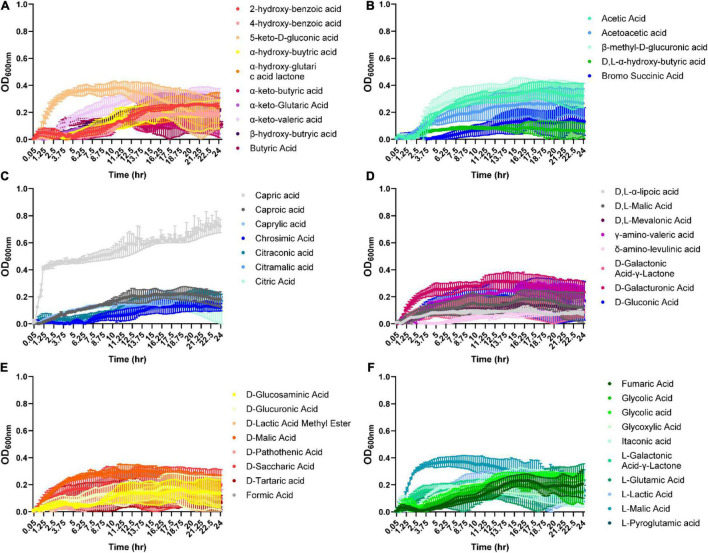
*Bacteroides ovatus* grows on certain acids in the absence of glucose. *B. ovatus* ATCC 8384 was grown anaerobically at 37°C in Biolog plates with a chemically-defined minimal media (CDMM) preparation that lacked glucose. Growth was monitored over 24 h by plate reader in plate containing various acids **(A–F)**. All data are presented as mean ± stdev.

**FIGURE 5:**
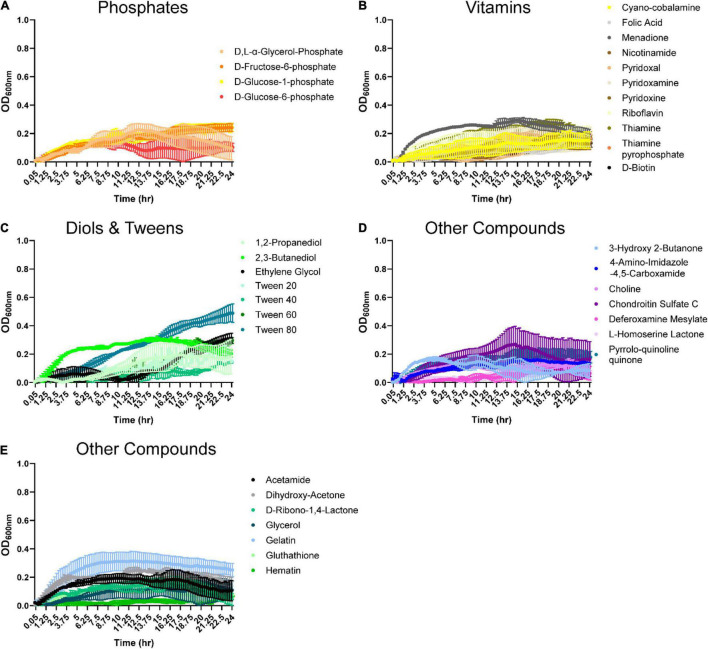
*Bacteroides ovatus* uses select substrates to support its growth. *B. ovatus* ATCC 8384 was grown anaerobically at 37°C in Biolog plates with a chemically-defined minimal media (CDMM) preparation that lacked glucose. Growth was monitored over 24 h by plate reader in plates containing **(A)** phosphates, **(B)** vitamins, **(C)** diols and tweens, and **(D,E)** other compounds. All data are presented as mean ± stdev.

Organic acids are commonly found in foods and can be produced by various microbes. Therefore, we examined *B. ovatus* growth in the presence of 48 different organic acids. Remarkably, *B. ovatus* was capable of using 2-hydroxy-benzoic acid, α-keto-valeric acid, β-hydroxy-butyric acid (Figure 4A), acetic acid, acetoacetic acid, β-methyl-D-glucuronic acid (Figure 4B and Supplementary Figure 1D), caproic acid, caprylic acid, citraconic acid (Figure 4C), γ-amino-valeric acid, D-galacturonic acid (Figure 4D), D-malic acid (Figure 4E), glycolic acid, glycoxylic acid, and L-malic acid (Figure 4F) for growth. Finally, we examined phosphates, vitamins, diols, tweens, and other compounds common to the intestinal environment (Figure 5 and Supplementary Figure 1E). *B. ovatus* was unable to use phosphates (Figure 5A) or vitamins (Figure 5B) to support its growth. However, *B. ovatus* was able to grow with the diols 2,3-butanediol and ethylene glycol, as well as tween 80 (Figure 5C). Additionally, *B. ovatus* was able to use chondroitin sulfate C (Figure 5D), dihydroxy-acetone, and gelatin (Figure 5E), indicating that *B. ovatus* is a highly versatile organism in terms of nutrient utilization.

**FIGURE 6:**
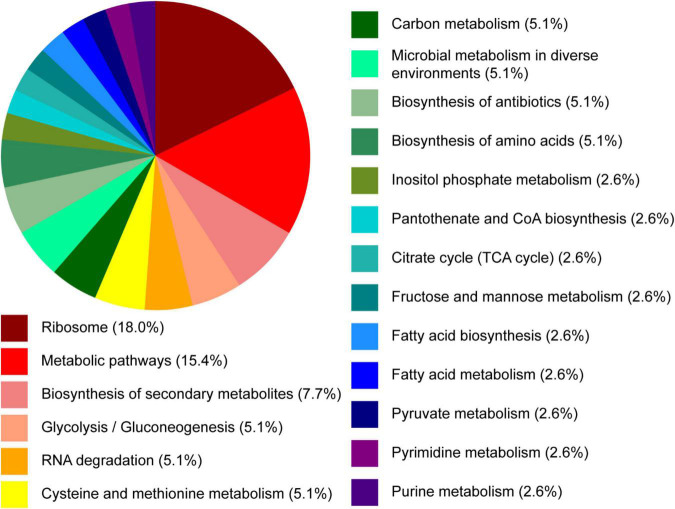
Proteomic pathway analysis of *B. ovatus* by proteomic analysis. *B. ovatus* ATCC 8384 was examined using high-resolution liquid chromatography-tandem mass spectrometry based proteomics. The functional classifications of these proteins are illustrated in the pie chart above.

**FIGURE 7:**
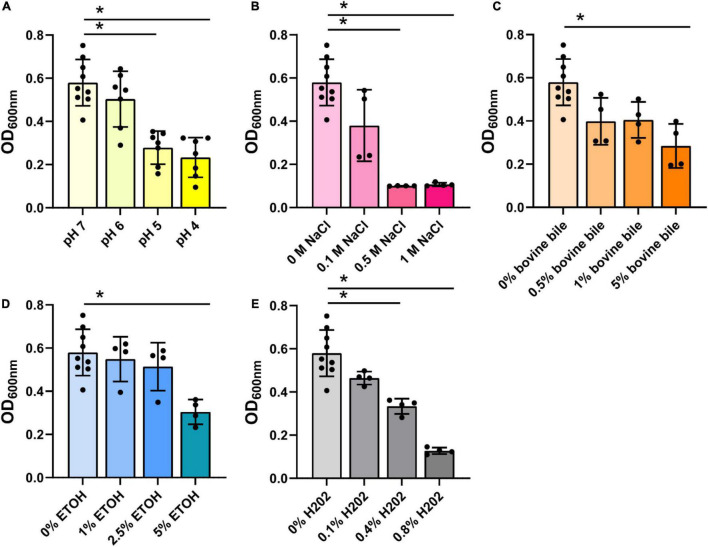
*Bacteroides ovatus* grows in conditions resembling the intestine. *B. ovatus* ATCC 8384 was grown anaerobically at 37°C in a chemically-defined minimal media (CDMM) preparation containing glucose and various **(A)** pHs, **(B)** NaCl concentrations (M), **(C)** bovine bile (%), **(D)** ethanol (%), and **(E)** hydrogen peroxide (%). All data are presented as mean ± stdev at the 24 h time point. *P < 0.05.

Next, we assessed the *B. ovatus* proteome. Using LC-MS/MS, we identified that 15.4% of the *B. ovatus* ATCC 8384 proteome was involved in metabolic pathways (Figure 6). These proteins included α-glucan phosphorylase (storage of polysaccharides), glucosamine-6-phosphate deaminase (generation of fructose 6-phosphate), galactokinase (phosphorylation of α-D-galactose), and others. We also identified proteins related to the metabolism of cysteine/methionine (5.1%), carbon (5.1%), fructose/mannose (2.6%), fatty acids (2.6%), pyruvate (2.6%), pyrimidine (2.6%), and purine (2.6%). Consistent with the ability to use diverse nutrient sources, we identified several glycosyl hydrolase (GH) proteins, including GH2, GH5, GH20, GH32, GH43, GH88, GH92, and GH95 (Table 1). We observed that 5.1% of the *B. ovatus* proteome corresponded with metabolism in diverse environments (Figure 6). We found several oxidative stress related proteins like thioredoxin reductase, antioxidant AhpC/TSA, thiamine pyrophosphate enzyme, *S*-adenosylmethionine synthase, oxidoreductase, and rubrerythrin. We predicted that the presence of these proteins may help *B. ovatus* survive in the gastrointestinal tract, which varies in terms of pH, osmolarity, bile, *etc*. To mirror stressors found in the gastrointestinal tract, we grew *B. ovatus* in CDMM containing glucose with varying pH, NaCl, bile, ethanol, and hydrogen peroxide concentration (Figure 7). *B. ovatus* grew well in CDMM at pH 7 and 6; similar to the intestinal pH range of 6–7 (Figure 7A). Varying NaCl concentrations were tested to mimic osmolarity fluctuations and we found that concentrations up to 0.1 M NaCl did not affect the ability of *B. ovatus* to grow (Figure 7B). However, NaCl values of 0.5 M and 1 M, which are outside the normal osmolarity range of the intestine, inhibited *B. ovatus* growth. Interestingly, *B. ovatus* maintained growth in 0.5 and 1% bovine bile but exhibited reduced growth with 5% bile (Figure 7C). Likewise, we found that B. ovatus tolerated 1 and 2.5% ethanol but was inhibited by 5% ethanol (Figure 7D). Finally, we observed no changes in *B. ovatus* growth with 0.1% H202 but decreasing levels of growth with 0.4 and 0.8% H202. Collectively these data indicate that *B. ovatus* is well equipped to sustain growth using diverse nutrient sources and represents a well-adapted gut commensal.

**TABLE 1:** Glycosyl hydrolases identified in *B. ovatus* ATCC 8384 by proteomic analysis.

Family	Accession	Description	Activities in Family	Function
GH2	A0A515IYC1	Glycoside hydrolase family 2 protein	β-galactosidase (EC 3.2.1.23); β-mannosidase (EC 3.2.1.25); β-glucuronidase (EC 3.2.1.31); α-L-arabinofuranosidase (EC 3.2.1.55); mannosylglycoprotein endo-β-mannosidase (EC 3.2.1.152); exo-β-glucosaminidase (EC 3.2.1.165); α-L-arabinopyranosidase (EC 3.2.1.-); β-galacturonidase (EC 3.2.1.-); β-xylosidase (EC 3.2.1.37); β-D-galactofuranosidase (EC 3.2.1.146); β-glucosidase	Hydrolase activity, hydrolyzing *O*-glycosyl compounds
GH5	A7LXR4	Cellulase (Glycosyl hydrolase family 5)	Endo-β-1,4-glucanase/cellulase (EC 3.2.1.4); endo-β-1,4-xylanase (EC 3.2.1.8); β-glucosidase (EC 3.2.1.21); β-mannosidase (EC 3.2.1.25); β-glucosylceramidase (EC 3.2.1.45); glucan β-1,3-glucosidase (EC 3.2.1.58); exo-β-1,4-glucanase/cellodextrinase (EC 3.2.1.74); glucan endo-1,6-β-glucosidase (EC 3.2.1.75); mannan endo-β-1,4-mannosidase (EC 3.2.1.78); cellulose β-1,4-cellobiosidase (EC 3.2.1.91); steryl β-glucosidase (EC 3.2.1.104); endoglycoceramidase (EC 3.2.1.123); chitosanase (EC 3.2.1.132); β-primeverosidase (EC 3.2.1.149); xyloglucan-specific endo-β-1,4-glucanase (EC 3.2.1.151); endo-β-1,6-galactanase (EC 3.2.1.164); β-1,3-mannanase (EC 3.2.1.-); arabinoxylan-specific endo-β-1,4-xylanase (EC 3.2.1.-); mannan transglycosylase (EC 2.4.1.-); lichenase/endo-β-1,3-1,4-glucanase (EC 3.2.1.73); β-glycosidase (EC 3.2.1.-); endo-β-1,3-glucanase/laminarinase (EC 3.2.1.39); β-*N*-acetylhexosaminidase (EC 3.2.1.52); chitosanase (EC 3.2.1.132); β-D-galactofuranosidase (EC 3.2.1.146); β-galactosylceramidase (EC 3.2.1.46); β-rutinosidase /α-L-rhamnose-(1,6)-β-D-glucosidase (EC 3.2.1.-); α-L-arabinofuranosidase (EC 3.2.1.55); glucomannan-specific endo-Î^2^-1,4-glucanase	Hydrolase activity, hydrolyzing *O*-glycosyl compounds
GH20	A0A5M5D8K1	Family 20 glycosylhydrolase	β-hexosaminidase (EC 3.2.1.52); lacto-*N*-biosidase (EC 3.2.1.140); β-1,6-*N*-acetylglucosaminidase (EC 3.2.1.-); β-6-SO3-*N*-acetylglucosaminidase (EC 3.2.1.-)	Hydrolysis of terminal non-reducing *N*-acetyl-D-hexosamine residues in *N*-acetyl-beta-D-hexosaminides
GH32	A0A413VC74	Glycoside hydrolase family 32 protein	Invertase (EC 3.2.1.26); endo-inulinase (EC 3.2.1.7); β-2,6-fructan 6-levanbiohydrolase (EC 3.2.1.64); endo-levanase (EC 3.2.1.65); exo-inulinase (EC 3.2.1.80); fructan β-(2,1)-fructosidase/1-exohydrolase (EC 3.2.1.153); fructan β-(2,6)-fructosidase/6-exohydrolase (EC 3.2.1.154); sucrose:sucrose 1-fructosyltransferase (EC 2.4.1.99); fructan:fructan 1-fructosyltransferase (EC 2.4.1.100); sucrose:fructan 6-fructosyltransferase (EC 2.4.1.10); fructan:fructan 6G-fructosyltransferase (EC 2.4.1.243); levan fructosyltransferase (EC 2.4.1.-); [retaining] sucrose:sucrose 6-fructosyltransferase (6-SST) (EC 2.4.1.-); cycloinulo-oligosaccharide fructanotransferase (EC 2.4.1.-)	Hydrolase activity, hydrolyzing *O*-glycosyl compounds
GH43	A0A515ITK9	Family 43 glycosyl hydrolase	β-xylosidase (EC 3.2.1.37); α-L-arabinofuranosidase (EC 3.2.1.55); xylanase (EC 3.2.1.8); α-1,2-L-arabinofuranosidase (EC 3.2.1.-); exo-α-1,5-L-arabinofuranosidase (EC 3.2.1.-); [inverting] exo-α-1,5-L-arabinanase (EC 3.2.1.-); β-1,3-xylosidase (EC 3.2.1.-); [inverting] exo-α-1,5-L-arabinanase (EC 3.2.1.-); [inverting] endo-α-1,5-L-arabinanase (EC 3.2.1.99); exo-β-1,3-galactanase (EC 3.2.1.145); β-D-galactofuranosidase (EC 3.2.1.146)	Hydrolase activity, hydrolyzing *O*-glycosyl compounds
GH43	A0A5M5M352	Family 43 glycosylhydrolase	β-xylosidase (EC 3.2.1.37); α-L-arabinofuranosidase (EC 3.2.1.55); xylanase (EC 3.2.1.8); α-1,2-L-arabinofuranosidase (EC 3.2.1.-); exo-α-1,5-L-arabinofuranosidase (EC 3.2.1.-); [inverting] exo-α-1,5-L-arabinanase (EC 3.2.1.-); β-1,3-xylosidase (EC 3.2.1.-); [inverting] exo-α-1,5-L-arabinanase (EC 3.2.1.-); [inverting] endo-α-1,5-L-arabinanase (EC 3.2.1.99); exo-β-1,3-galactanase (EC 3.2.1.145); β-D-galactofuranosidase (EC 3.2.1.146)	Hydrolase activity, hydrolyzing *O*-glycosyl compounds
GH88	A0A5M5NKV7	Glycosyl hydrolase family 88	D-4,5-unsaturated β-glucuronyl hydrolase (EC 3.2.1.-)	hydrolase activity
GH88	A0A5M5EPT8	Glucuronyl hydrolase	D-4,5-unsaturated β-glucuronyl hydrolase (EC 3.2.1.-)	hydrolase activity
GH92	A0A641WAJ0	Glycoside hydrolase family 92 protein	Mannosyl-oligosaccharide α-1,2-mannosidase (EC 3.2.1.113); mannosyl-oligosaccharide α-1,3-mannosidase (EC 3.2.1.-); mannosyl-oligosaccharide α-1,6-mannosidase (EC 3.2.1.-); α-mannosidase (EC 3.2.1.24); α-1,2-mannosidase (EC 3.2.1.-); α-1,3-mannosidase (EC 3.2.1.-); α-1,4-mannosidase (EC 3.2.1.-); mannosyl-1-phosphodiester α-1,P-mannosidase (EC 3.2.1.-)	Hydrolase activity
GH92	A0A5M5N3X7	Glycoside hydrolase family 92 protein	Mannosyl-oligosaccharide α-1,2-mannosidase (EC 3.2.1.113); mannosyl-oligosaccharide α-1,3-mannosidase (EC 3.2.1.-); mannosyl-oligosaccharide α-1,6-mannosidase (EC 3.2.1.-); α-mannosidase (EC 3.2.1.24); α-1,2-mannosidase (EC 3.2.1.-); α-1,3-mannosidase (EC 3.2.1.-); α-1,4-mannosidase (EC 3.2.1.-); mannosyl-1-phosphodiester α-1,*P*-mannosidase (EC 3.2.1.-)	Hydrolase activity
GH95	A0A641QB35	Glycoside hydrolase family 95 protein	α-L-fucosidase (EC 3.2.1.51); α-1,2-L-fucosidase (EC 3.2.1.63); α-L-galactosidase (EC 3.2.1.-)	Alpha-L-fucosidase activity

## Discussion

**FIGURE 8:**
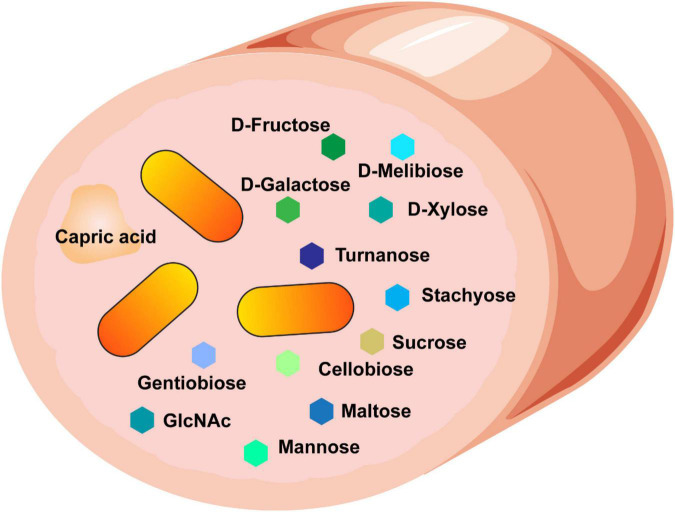
Our data suggest that *B. ovatus* consumes a variety of dietary sources and we speculate that these features contribute to the ability of *B. ovatus* to colonize the intestine.

We found that *B. ovatus* is a versatile gut microbe in terms of nutrient utilization and management of environmental stressors. We identified that *B. ovatus* ATCC 8384 can use 10 disaccharides, 4 trisaccharides, 4 polysaccharides, 4 polymers, 3 L-linked sugars, 6 D-linked sugars, 5 amino-sugars, 6 alcohol sugars, and 15 organic acids for growth (Figure 8). While not an exhaustive list, this impressive number of nutrient sources represents a wide range of options to support the growth of *B. ovatus* in an environment that is ever changing like the GI tract. The ability to use select nutrient sources was likewise reflected by the glycosyl hydrolases present in the proteome of *B. ovatus* ATCC 8384. Finally, we found that *B. ovatus* ATCC 8384 grew in conditions that mirrored the intestine, highlighting its role as a commensal gut microbe.

The gut microbiota contributes substantially to host health. Commensal gut microbes are known to modulate the immune system, enhance the epithelial barrier, prevent pathogen colonization, and provide key nutrients (Engevik and Versalovic, 2017). The importance of these microbes is highlighted by the fact that alterations in microbial community structure, or dysbiosis, are associated with numerous diseases, including inflammatory bowel, metabolic, cardiovascular, and neurological diseases. As a result, controlling the composition of the intestinal microbiota is an important factor in maintaining human health. Studies in human subjects have revealed that diet plays a significant role in shaping the microbiota and dietary alterations can induce large microbial shifts within 24 h (Singh et al., 2017). Bacteroides are a dominant bacterial genus in the healthy adult microbiota. Of the members of genus Bacteroides, *B. ovatus* has recently garnered attention for its anti-inflammatory and health-promoting properties (Hudcovic et al., 2009; Ihekweazu et al., 2019, 2021; Tan et al., 2019). The potential to shape the composition of Bacteroides, particularly *B. ovatus*, within the human gastrointestinal tract with specific combinations of dietary nutrients merits further study.

Our data revealed that *B. ovatus* ATCC 8384 could use multiple L- and D-linked sugars as growth substrates. However, the majority of dietary monosaccharides are absorbed in the small intestine and are unlikely to be found in the colon; the primary site of *B. ovatus* colonization. In the colon, *B. ovatus* likely encounters complex polysaccharides which are not host digestible. These likely include plant cell wall polysaccharides (dietary fiber), animal polysaccharides, plant and animal glycoproteins, natural and derivatized polysaccharides used as food rheology modifiers, and oligosaccharides. Our data also shows that *B. ovatus* can use multiple trisaccharides, polysaccharides, and polymers. For example, we found that *B. ovatus* can use inulin for growth. Inulin is a naturally occurring polysaccharide mixture produced by several plants and commonly found in industrialized foods as a fat substitute. Inulin is also considered a prebiotic or non-digestible food ingredient (Abed et al., 2016). Other groups have noted the utilization of inulin by *Bacteroides* spp. including *B. ovatus* (Sonnenburg et al., 2010). Rakoff-Nahoum et al. (2016) found that digestion of inulin *in vitro* and *in vivo* increased the fitness of *B. ovatus* due to reciprocal cross-feeding with another species *B. vulgatus*. Additionally, in a randomized controlled trial, patients consuming inulin and oligofructose had a significant increase in fecal *B. ovatus* levels (Birkeland et al., 2020); indicating that diet can significantly influence *B. ovatus in vivo*.

We also found that *B. ovatus* grew well with another prebiotic fiber, pectin. Consistent with our work, Chung et al. (2016) found that the addition of inulin and pectin to human gut microbial communities cultured in anaerobic continuous flow fermenters increased Bacteroides abundance, and specifically *B. ovatus*. In another study using *in vitro* fermenters, Larsen et al. observed that *B. ovatus* increased in concentrations with diverse pectins (Larsen et al., 2019). Using a defined microbial community of five species, another group found that *B. ovatus* increased in growth with pectin (Liu et al., 2020). Work by Pudlo et al. (2021) identified that multiple *B. ovatus* strains could use pectins, starch, fructans, and glycosaminoglycans. These findings, as well as our own, indicate that the addition of polysaccharides like inulin and pectin to the diet could promote *B. ovatus* growth and potentially increase *B. ovatus* levels in the colon.

To degrade complex substrates, gut microbes can encode a vast array of carbohydrate-active enzymes (CAZymes) and associated proteins to forage nutrients in the gut. In our proteome analysis, we observed multiple glycosyl hydrolases involved in the metabolism of dietary substances. *B. ovatus* ATCC 8384 possessed GH2, GH5 and GH92; families which harbor α- and β-mannosidases. In our growth analysis, we found that *B. ovatus* was able to use mannan to support its growth (Figure 1E) and we speculate that this might be due to GH2, GH5 or GH92 mannosidases. GH2 and GH5 also contain β-glucosidases, which may help explain the ability of *B. ovatus* to use cellobiose as a substrate (Figure 1C). The *B. ovatus* genome contains multiple gene copies of GH43 (β-xylosidases) and we observed the presence of a GH43 protein in our proteomic analysis. β-xylosidases hydrolyze xyloglucan, a component of plant cell wall polysaccharides, into single xylose units. Although xyloglucan was not included in our microarrays, previous groups have shown that *B. ovatus* possesses a cell-surface endo-glucanase, which coordinates xyloglucan uptake, and a xyloglucan utilization locus (XyGUL; comprising α-xylosidase, a β-glucosidase, and two α-l-arabinofuranosidases), which promotes the growth of *B. ovatus* on xyloglucan and xylooligosaccharides (Hemsworth et al., 2016; Tauzin et al., 2016; Mendis et al., 2018; Foley et al., 2019). Another study found that *B. ovatus* was the only *Bacteroides* spp. that could grow in minimal medium containing arabinoxylan as the sole carbon source (Wu et al., 2015). Importantly, *B. ovatus* ATCC 8483 has been shown to grow on xylan isolated from a variety of plants (Centanni et al., 2017), highlighting once again the importance of diet in the manipulation of *B. ovatus* levels.

While the use of polysaccharides and carbohydrates by the gut microbiota have been well documented, the digestive fate of amino acids and other dietary organic acids are less well understood. Our data indicate that *B. ovatus* can only nominally use amino acids for growth, indicating that a carbon source is necessary for proper growth. Interestingly, we found that several organic acids could promote growth to OD_600__nm_ > 0.4. For example, we found that *B. ovatus* could use capric acid, a medium-chain fatty acid found in saturated fats, coconut oil, palm kernel oil, cow’s milk, and goat’s milk. We also observed that *B. ovatus* could grow with malic acid; a dicarboxylic acid that contributes to the sour taste of fruits and is commonly used as a food additive. These substrates may represent unexplored nutrient niches for commensal microbes and therefore help mediate colonization. By using a minimal fully defined media, we were able to identify novel compounds, such as organic acids, which stimulated *B. ovatus* growth. However, it is possible that some compounds work in synergy. Future studies using richer media would help define the interplay between nutrient sources in *B. ovatus*.

In summary, our data provides a deeper, more comprehensive profile of nutrient utilization by the commensal microbe *B. ovatus* ATCC 8384. We hope that our characterization will be incorporated into existing metabolic models for *B. ovatus*. Our data demonstrate that *B. ovatus* is extremely versatile in terms of nutrient utilization and commonly used prebiotics, as well as potentially unique dietary sources such as food-related organic acids. With this information, we envision designing specific diets based on nutrient components targeted at modulating *B. ovatus* levels in the gut. Further experiments using animal models will be necessary to confirm the ability of *B. ovatus* to use these dietary sources to drive colonization, but in the future, we think it might be possible to stimulate *B. ovatus* growth to promote long-lasting health effects in the host.

## Data Availability Statement

The datasets presented in this study can be found in online repositories. The names of the repository/repositories and accession number(s) can be found below: https://massive.ucsd.edu/ProteoSAFe/private-dataset.jsp?task=9e9343b12d3540e7bc8c470f2de719d3, MSV000086294.

## Author Contributions

RF, SB, and ME: concept and design. RF, TT, FI, TH, SH, KH, MB, JS, AH, SB, and ME: intellectual contribution. RF, TT, FI, TH, SH, and KH: data acquisition. RF, TT, FI, TH, SH, KH, MB, JS, AH, SB, and ME: data analysis, statistics, and interpretation. RF and ME: drafting manuscript. RF, TT, FI, TH, SH, KH, MB, JS, AH, SB, and ME: editing manuscript. AH, SB, and ME: funding. All authors: contributed to the article and approved the submitted version.

## Conflict of Interest

JS receives unrestricted research support from Biogaia, AB. The remaining authors declare that the research was conducted in the absence of any commercial or financial relationships that could be construed as a potential conflict of interest.

## Publisher’s Note

All claims expressed in this article are solely those of the authors and do not necessarily represent those of their affiliated organizations, or those of the publisher, the editors and the reviewers. Any product that may be evaluated in this article, or claim that may be made by its manufacturer, is not guaranteed or endorsed by the publisher.
