# Supplementary material for: Unraveling the Metabolic Requirements of the Gut Commensal Bacteroides ovatus
Source: Front Microbiol. 2021 Nov 25;12:745469. doi: 10.3389/fmicb.2021.745469 (PMC8656163; doi:10.3389/fmicb.2021.745469)
Supplement: Supplementary file 1 [file Data_Sheet_1.docx]

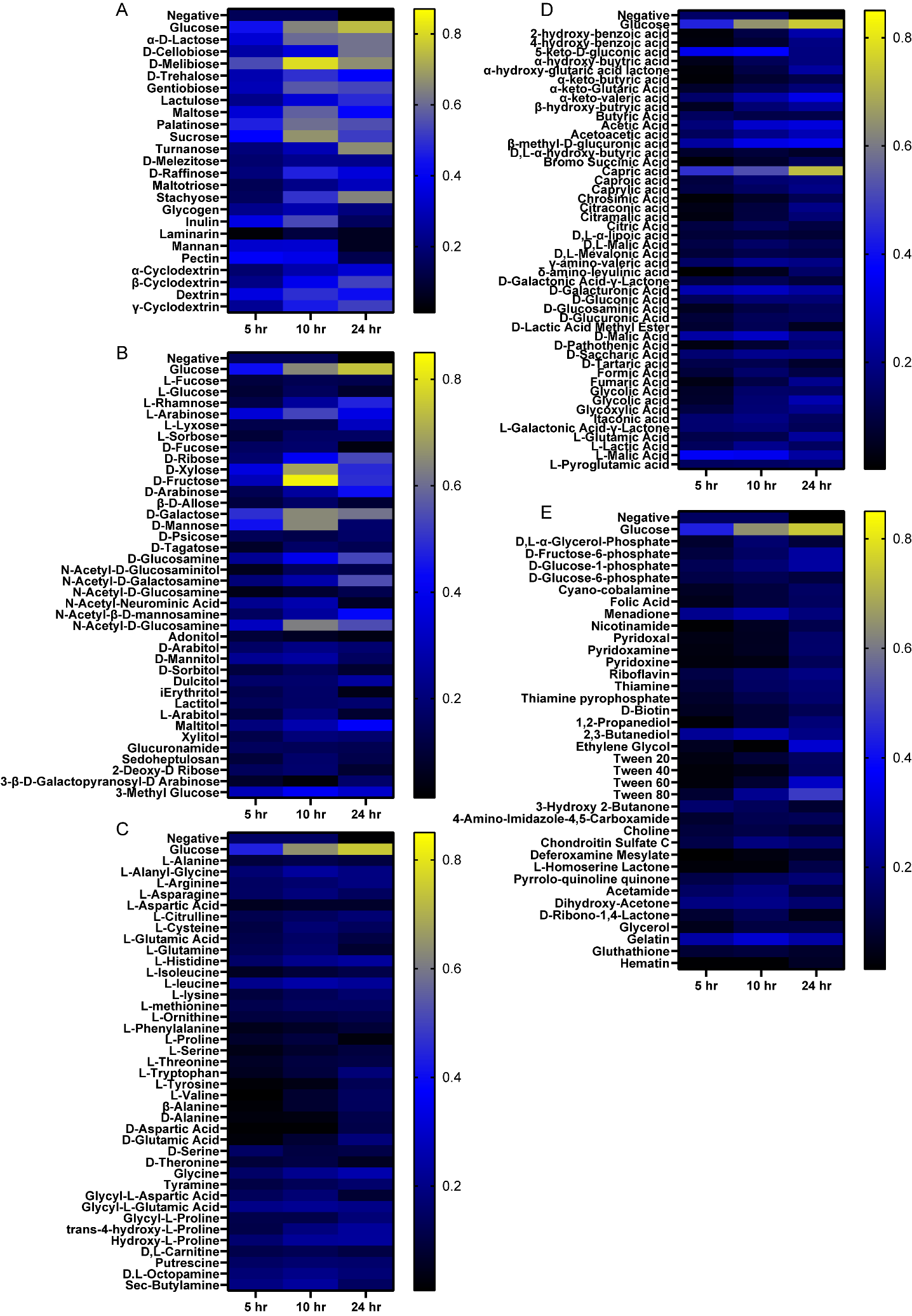


**Supplemental Figure 1**. For visualization, heat maps were generated for (**A**) disaccharides, trisaccharides, polysacchrides, and polymers, (**B**) sugars, (**C**) amino acids, (**D**) acids, and (**E**) phosphates, vitamins, diols, tweens, and other compounds at time 5, 10 and 24 hr.
