# Supplementary material for: Unraveling the Metabolic Requirements of the Gut Commensal Bacteroides ovatus
Source: Front Microbiol. 2021 Nov 25;12:745469. doi: 10.3389/fmicb.2021.745469 (PMC8656163; doi:10.3389/fmicb.2021.745469)
Supplement: Supplementary file 2 [file Table_1.docx]

**Supplemental Table 1:** Statistics from growth curves at time point 0, 5, 10 and 24 hours. Significant p values are denoted as P<0.05 and are highlighted in blue.

| **Controls** |  |  |  |  |
| --- | --- | --- | --- | --- |
| **Comparison** | **0 hr** | **5 hr** | **10 hr** | **24 hr** |
| Negative vs. Glucose | 0.9997 | 0.0029 | <0.0001 | <0.0001 |
| **Disaccharides** |  |  |  |  |
| **Comparison** | **0 hr** | **5 hr** | **10 hr** | **24 hr** |
| Negative vs. α-D-Lactose | >0.9999 | 0.5273 | 0.0002 | <0.0001 |
| Negative vs. D-Cellobiose | >0.9999 | 0.9701 | 0.4048 | <0.0001 |
| Negative vs. D-Melibiose | >0.9999 | 0.0021 | <0.0001 | <0.0001 |
| Negative vs. D-Trehalose | >0.9999 | 0.9189 | 0.0094 | 0.012 |
| Negative vs. Gentiobiose | >0.9999 | 0.8704 | 0.0009 | 0.0001 |
| Negative vs. Lactulose | >0.9999 | 0.9994 | 0.4619 | 0.0007 |
| Negative vs. Maltose | >0.9999 | 0.4978 | 0.0005 | 0.0076 |
| Negative vs. Palatinose | 0.9999 | 0.0285 | 0.0002 | <0.0001 |
| Negative vs. Sucrose | >0.9999 | 0.1289 | <0.0001 | 0.0002 |
| Negative vs. Turnanose | >0.9999 | 0.9998 | 0.8622 | <0.0001 |
| **Trisaccharide’s** |  |  |  |  |
| **Comparison** | **0 hr** | **5 hr** | **10 hr** | **24 hr** |
| Negative vs. D-Melezitose | 0.9999 | >0.9999 | 0.2753 | 0.05 |
| Negative vs. D-Raffinose | 0.9999 | 0.999 | 0.0025 | 0.0074 |
| Negative vs. Maltotriose | 0.9999 | 0.9622 | 0.9524 | 0.0365 |
| Negative vs. Stachyose | >0.9999 | 0.9896 | 0.0008 | <0.0001 |
| **Polysaccharides** |  |  |  |  |
| **Comparison** | **0 hr** | **5 hr** | **10 hr** | **24 hr** |
| Negative vs. Glycogen | >0.9999 | 0.8813 | 0.049 | 0.1281 |
| Negative vs. Inulin | 0.9982 | 0.0038 | <0.0001 | 0.6521 |
| Negative vs. Laminarin | 0.996 | 0.0117 | 0.5247 | 0.9522 |
| Negative vs. Mannan | >0.9999 | 0.0263 | 0.0166 | 0.9479 |
| Negative vs. Pectin | >0.9999 | 0.0008 | 0.002 | 0.9325 |
| **Polymers** |  |  |  |  |
| **Comparison** | **0 hr** | **5 hr** | **10 hr** | **24 hr** |
| Negative vs. α-Cyclodextrin | 0.9925 | >0.9999 | 0.2005 | 0.0001 |
| Negative vs. β-Cyclodextrin | 0.9985 | 0.9137 | 0.0019 | <0.0001 |
| Negative vs. Dextrin | 0.9742 | 0.006 | <0.0001 | <0.0001 |
| Negative vs. γ-Cyclodextrin | 0.9978 | 0.6504 | <0.0001 | <0.0001 |
| **L-linked sugars** |  |  |  |  |
| **Comparison** | **0 hr** | **5 hr** | **10 hr** | **24 hr** |
| Negative vs. L-Fucose | >0.9999 | 0.7626 | 0.9815 | 0.9789 |
| Negative vs. L-Glucose | >0.9999 | 0.5929 | 0.9983 | 0.9997 |
| Negative vs. L-Rhamnose | >0.9999 | 0.8753 | 0.6943 | <0.0001 |
| Negative vs. L-Arabinose | 0.9999 | 0.0537 | <0.0001 | 0.0004 |
| Negative vs. L-Lyxose | >0.9999 | 0.9356 | 0.9602 | 0.0056 |
| Negative vs. L-Sorbose | >0.9999 | 0.6499 | 0.9998 | 0.7307 |
| **D-linked sugars** |  |  |  |  |
| **Comparison** | **0 hr** | **5 hr** | **10 hr** | **24 hr** |
| Negative vs. D-Fucose | >0.9999 | 0.9997 | >0.9999 | 0.9931 |
| Negative vs. D-Ribose | >0.9999 | >0.9999 | 0.0609 | <0.0001 |
| Negative vs. D-Xylose | 0.9999 | 0.1821 | <0.0001 | <0.0001 |
| Negative vs. D-Fructose | >0.9999 | 0.6474 | <0.0001 | <0.0001 |
| Negative vs. D-Arabinose | >0.9999 | 0.9974 | 0.9373 | 0.0005 |
| Negative vs. β-D-Allose | 0.9999 | 0.9719 | >0.9999 | >0.9999 |
| Negative vs. D-Galactose | >0.9999 | 0.0019 | <0.0001 | <0.0001 |
| Negative vs. D-Mannose | 0.9999 | 0.0143 | <0.0001 | 0.9159 |
| Negative vs. D-Psicose | >0.9999 | 0.9995 | 0.9969 | 0.9466 |
| Negative vs. D-Tagatose | >0.9999 | 0.7885 | 0.9999 | 0.9968 |
| **Amino sugars** |  |  |  |  |
| **Comparison** | **0 hr** | **5 hr** | **10 hr** | **24 hr** |
| Negative vs. D-Glucosamine | 0.9999 | 0.7285 | 0.0004 | <0.0001 |
| Negative vs. N-Acetyl-D-Glucosaminitol | >0.9999 | 0.0564 | 0.9974 | 0.946 |
| Negative vs. N-Acetyl-D-Galactosamine | 0.9996 | 0.9996 | 0.1885 | <0.0001 |
| Negative vs. N-Acetyl-D-Glucosamine | >0.9999 | 0.0357 | 0.2864 | 0.9139 |
| Negative vs. N-Acetyl-Neurominic Acid | >0.9999 | 0.7954 | 0.1434 | 0.9953 |
| Negative vs. N-Acetyl-β-D-mannosamine | >0.9999 | 0.9977 | 0.4124 | <0.0001 |
| Negative vs. N-Acetyl-D-Glucosamine | 0.9998 | 0.0409 | <0.0001 | <0.0001 |
| **Alcohol sugars** |  |  |  |  |
| **Comparison** | **0 hr** | **5 hr** | **10 hr** | **24 hr** |
| Negative vs. D-Sorbitol | >0.9999 | 0.2566 | 0.9896 | 0.9997 |
| Negative vs. Dulcitol | >0.9999 | 0.9998 | >0.9999 | 0.0003 |
| Negative vs. iErythritol | 0.9999 | 0.7409 | 0.9999 | 0.739 |
| Negative vs. Lactitol | >0.9999 | 0.9487 | 0.9997 | 0.0954 |
| Negative vs. L-Arabitol | 0.9999 | 0.3489 | 0.9813 | 0.9997 |
| Negative vs. Maltitol | >0.9999 | 0.9918 | 0.0349 | <0.0001 |
| Negative vs. Xylitol | 0.9999 | 0.6094 | 0.991 | 0.0922 |
| **Other sugars** |  |  |  |  |
| **Comparison** | **0 hr** | **5 hr** | **10 hr** | **24 hr** |
| Negative vs. Glucuronamide | >0.9999 | 0.9672 | 0.9548 | 0.7219 |
| Negative vs. Sedoheptulosan | 0.9999 | 0.2458 | >0.9999 | 0.8561 |
| Negative vs. 2-Deoxy-D Ribose | 0.9998 | 0.9472 | 0.9999 | 0.9999 |
| Negative vs. 3-β-D-Galactopyranosyl-D Arabinose | 0.9944 | 0.0753 | 0.0052 | 0.1912 |
| Negative vs. 3-Methyl Glucose | >0.9999 | 0.0341 | <0.0001 | <0.0001 |
| **L-amino acids** |  |  |  |  |
| **Comparison** | **0 hr** | **5 hr** | **10 hr** | **24 hr** |
| Negative vs. L-Alanine | 0.9997 | 0.2725 | 0.7558 | 0.9999 |
| Negative vs. L-Alanyl-Glycine | >0.9999 | 0.9995 | 0.9241 | 0.1819 |
| Negative vs. L-Arginine | >0.9999 | 0.9938 | 0.9995 | 0.1895 |
| Negative vs. L-Asparagine | 0.9999 | 0.9945 | 0.9999 | 0.9556 |
| Negative vs. L-Aspartic Acid | 0.9994 | 0.0189 | 0.0854 | 0.9991 |
| Negative vs. L-Citrulline | 0.9998 | 0.7558 | 0.9946 | 0.4536 |
| Negative vs. L-Cysteine | 0.9991 | 0.4221 | 0.9997 | 0.9758 |
| Negative vs. L-Glutamic Acid | 0.9997 | 0.6364 | 0.999 | 0.9996 |
| Negative vs. L-Glutamine | >0.9999 | 0.7914 | 0.9993 | 0.9993 |
| Negative vs. L-Histidine | >0.9999 | 0.999 | 0.9989 | 0.0125 |
| Negative vs. L-Isoleucine | 0.9995 | 0.0266 | 0.2698 | 0.9994 |
| Negative vs. L-leucine | 0.9999 | 0.9991 | 0.6704 | 0.022 |
| Negative vs. L-lysine | 0.9997 | 0.2933 | 0.9671 | 0.6294 |
| Negative vs. L-methionine | 0.9997 | 0.7175 | 0.9945 | 0.9508 |
| Negative vs. L-Ornithine | 0.9999 | 0.3399 | 0.5371 | 0.9992 |
| Negative vs. L-Phenylalanine | 0.9994 | 0.0115 | 0.0571 | 0.9998 |
| Negative vs. L-Proline | 0.9998 | 0.0673 | 0.3689 | 0.687 |
| Negative vs. L-Serine | 0.9998 | 0.0069 | 0.098 | >0.9999 |
| Negative vs. L-Threonine | 0.9998 | 0.0364 | 0.4041 | 0.9996 |
| Negative vs. L-Tryptophan | 0.9997 | 0.0172 | 0.3147 | 0.333 |
| Negative vs. L-Tyrosine | 0.9997 | 0.0008 | 0.0069 | 0.9954 |
| Negative vs. L-Valine | 0.9998 | 0.0003 | 0.1224 | 0.9634 |
| **D-amino acids** |  |  |  |  |
| **Comparison** | **0 hr** | **5 hr** | **10 hr** | **24 hr** |
| Negative vs. β-Alanine | 0.9997 | <0.0001 | 0.0017 | 0.5012 |
| Negative vs. D-Alanine | 0.9994 | <0.0001 | <0.0001 | 0.9654 |
| Negative vs. D-Aspartic Acid | 0.9994 | <0.0001 | <0.0001 | 0.9917 |
| Negative vs. D-Glutamic Acid | 0.9995 | <0.0001 | 0.0048 | 0.0191 |
| Negative vs. D-Serine | >0.9999 | 0.7451 | 0.0472 | 0.9969 |
| Negative vs. D-Theronine | 0.9999 | 0.003 | 0.0907 | 0.7426 |
| Negative vs. Glycine | 0.9995 | 0.8458 | 0.809 | <0.0001 |
| Negative vs. Tyramine | >0.9999 | 0.0402 | 0.429 | 0.1159 |
| **Modified amino acids** |  |  |  |  |
| **Comparison** | **0 hr** | **5 hr** | **10 hr** | **24 hr** |
| Negative vs. Glycyl-L-Aspartic Acid | >0.9999 | 0.531 | 0.9997 | 0.9993 |
| Negative vs. Glycyl-L-Glutamic Acid | 0.9999 | 0.9966 | 0.7451 | 0.0059 |
| Negative vs. Glycyl-L-Proline | >0.9999 | 0.1734 | 0.1974 | 0.0569 |
| Negative vs. trans-4-hydroxy-L-Proline | 0.9997 | 0.1204 | 0.9996 | 0.0002 |
| Negative vs. Hydroxy-L-Proline | >0.9999 | 0.9965 | 0.5984 | 0.0002 |
| Negative vs. D,L-Carnitine | 0.9998 | 0.1723 | 0.4507 | 0.9995 |
| Negative vs. Putrescine | >0.9999 | 0.8426 | 0.9993 | 0.1734 |
| Negative vs. D.L-Octopamine | >0.9999 | 0.9996 | 0.9777 | 0.0719 |
| Negative vs. Sec-Butylamine | >0.9999 | 0.9995 | 0.4332 | 0.453 |
| **Acids** |  |  |  |  |
| **Comparison** | **0 hr** | **5 hr** | **10 hr** | **24 hr** |
| Negative vs. 2-hydroxy-benzoic acid | >0.9999 | 0.1583 | 0.9979 | 0.2754 |
| Negative vs. 4-hydroxy-benzoic acid | >0.9999 | 0.1932 | 0.8979 | 0.9478 |
| Negative vs. 5-keto-D-gluconic acid | >0.9999 | 0.1868 | 0.0435 | 0.9773 |
| Negative vs. α-hydroxy-buytric acid | >0.9999 | 0.3487 | 0.999 | 0.98 |
| Negative vs. α-hydroxy-glutaric acid lactone | 0.9999 | 0.1619 | 0.998 | 0.4497 |
| Negative vs. α-keto-butyric acid | >0.9999 | 0.1354 | 0.8661 | 0.9997 |
| Negative vs. α-keto-Glutaric Acid | >0.9999 | 0.7243 | 0.9986 | 0.9979 |
| Negative vs. α-keto-valeric acid | 0.9999 | 0.9994 | 0.9984 | 0.002 |
| Negative vs. β-hydroxy-butryic acid | >0.9999 | 0.5137 | 0.9998 | 0.6364 |
| Negative vs. Butyric Acid | >0.9999 | 0.9988 | 0.9993 | 0.9997 |
| Negative vs. Acetic Acid | >0.9999 | 0.9999 | 0.737 | 0.0061 |
| Negative vs. Acetoacetic acid | >0.9999 | 0.999 | 0.9996 | 0.1248 |
| Negative vs. β-methyl-D-glucuronic acid | >0.9999 | 0.999 | 0.2383 | 0.0006 |
| Negative vs. D,L-α-hydroxy-butyric acid | 0.9998 | 0.7382 | 0.9628 | 0.9994 |
| Negative vs. Bromo Succinic Acid | >0.9999 | 0.1274 | 0.7839 | 0.9993 |
| Negative vs. Capric acid | 0.9997 | 0.0002 | <0.0001 | <0.0001 |
| Negative vs. Caproic acid | >0.9999 | 0.9694 | 0.9997 | 0.9828 |
| Negative vs. Caprylic acid | 0.9999 | 0.9842 | 0.9993 | 0.9174 |
| Negative vs. Chrosimic Acid | 0.9999 | 0.1248 | 0.6743 | 0.9995 |
| Negative vs. Citraconic acid | >0.9999 | 0.303 | 0.9822 | 0.9071 |
| Negative vs. Citramalic acid | >0.9999 | 0.2134 | 0.9833 | 0.9981 |
| Negative vs. Citric Acid | >0.9999 | 0.9818 | 0.9992 | >0.9999 |
| Negative vs. D,L-α-lipoic acid | 0.9999 | 0.9167 | 0.9694 | 0.9997 |
| Negative vs. D,L-Malic Acid | >0.9999 | 0.9865 | 0.9994 | 0.9995 |
| Negative vs. D,L-Mevalonic Acid | 0.9999 | 0.924 | 0.9984 | 0.9999 |
| Negative vs. γ-amino-valeric acid | >0.9999 | 0.9993 | 0.9994 | 0.9312 |
| Negative vs. δ-amino-levulinic acid | >0.9999 | 0.0772 | 0.4868 | 0.9988 |
| Negative vs. D-Galactonic Acid-γ-Lactone | >0.9999 | 0.9814 | 0.9988 | 0.9999 |
| Negative vs. D-Galacturonic Acid | >0.9999 | 0.9983 | 0.928 | 0.4214 |
| Negative vs. D-Gluconic Acid | >0.9999 | 0.9989 | >0.9999 | 0.998 |
| Negative vs. D-Glucosaminic Acid | >0.9999 | 0.4812 | 0.998 | 0.9993 |
| Negative vs. D-Glucuronic Acid | >0.9999 | 0.9298 | 0.999 | 0.9992 |
| Negative vs. D-Lactic Acid Methyl Ester | >0.9999 | 0.8259 | 0.9988 | 0.9991 |
| Negative vs. D-Malic Acid | >0.9999 | 0.9988 | 0.8603 | 0.9588 |
| Negative vs. D-Pathothenic Acid | >0.9999 | 0.24 | 0.891 | 0.9985 |
| Negative vs. D-Saccharic Acid | 0.9998 | 0.9997 | 0.9996 | 0.8871 |
| Negative vs. D-Tartaric acid | 0.9999 | 0.998 | 0.9985 | 0.9995 |
| Negative vs. Formic Acid | >0.9999 | 0.9456 | 0.9996 | >0.9999 |
| Negative vs. Fumaric Acid | >0.9999 | 0.2549 | 0.998 | 0.799 |
| Negative vs. Glycolic Acid | >0.9999 | 0.6584 | 0.9995 | 0.999 |
| Negative vs. Glycolic acid | >0.9999 | 0.7109 | 0.9997 | 0.1812 |
| Negative vs. Glycoxylic Acid | >0.9999 | 0.9821 | 0.9999 | 0.7978 |
| Negative vs. Itaconic acid | 0.9999 | 0.9996 | 0.9998 | 0.9991 |
| Negative vs. L-Galactonic Acid-γ-Lactone | >0.9999 | 0.9996 | >0.9999 | 0.9994 |
| Negative vs. L-Glutamic Acid | 0.9997 | 0.9982 | 0.9985 | 0.498 |
| Negative vs. L-Lactic Acid | 0.9999 | 0.999 | 0.9995 | 0.9991 |
| Negative vs. L-Malic Acid | >0.9999 | 0.1048 | 0.139 | 0.4097 |
| Negative vs. L-Pyroglutamic acid | >0.9999 | 0.9991 | 0.9994 | 0.9986 |
| **Phosphates** |  |  |  |  |
| **Comparison** | **0 hr** | **5 hr** | **10 hr** | **24 hr** |
| Negative vs. D,L-α-Glycerol-Phosphate | 0.9998 | 0.0191 | 0.9965 | >0.9999 |
| Negative vs. D-Fructose-6-phosphate | 0.9998 | 0.094 | 0.9114 | 0.001 |
| Negative vs. D-Glucose-1-phosphate | >0.9999 | 0.4138 | >0.9999 | 0.0008 |
| Negative vs. D-Glucose-6-phosphate | 0.9999 | 0.1818 | 0.498 | 0.9986 |
| **Vitamins** |  |  |  |  |
| **Comparison** | **0 hr** | **5 hr** | **10 hr** | **24 hr** |
| Negative vs. Cyano-cobalamine | >0.9999 | 0.0389 | 0.3789 | 0.7207 |
| Negative vs. Folic Acid | >0.9999 | 0.0162 | 0.3753 | 0.8088 |
| Negative vs. Menadione | 0.9999 | 0.9924 | 0.3697 | 0.19 |
| Negative vs. Nicotinamide | 0.9999 | 0.0017 | 0.0497 | 0.9938 |
| Negative vs. Pyridoxal | 0.9997 | 0.0084 | 0.0407 | 0.5681 |
| Negative vs. Pyridoxamine | 0.9999 | 0.006 | 0.0453 | 0.5325 |
| Negative vs. Pyridoxine | 0.9998 | 0.0038 | 0.01 | 0.9256 |
| Negative vs. Riboflavin | 0.9998 | 0.4844 | 0.9994 | 0.1035 |
| Negative vs. Thiamine | 0.9999 | 0.1999 | 0.9961 | 0.3443 |
| Negative vs. Thiamine pyrophosphate | 0.9998 | 0.0726 | 0.7619 | 0.4448 |
| **Diols & Tweens** |  |  |  |  |
| **Comparison** | **0 hr** | **5 hr** | **10 hr** | **24 hr** |
| Negative vs. 1,2-Propanediol | >0.9999 | 0.0033 | 0.206 | 0.2472 |
| Negative vs. 2,3-Butanediol | >0.9999 | 0.8955 | 0.2023 | 0.2472 |
| Negative vs. Ethylene Glycol | >0.9999 | 0.0352 | 0.004 | <0.0001 |
| Negative vs. Tween 20 | 0.9998 | 0.0101 | 0.23 | 0.7277 |
| Negative vs. Tween 40 | >0.9999 | 0.0054 | 0.0162 | 0.8178 |
| Negative vs. Tween 60 | 0.9999 | 0.0044 | 0.1555 | 0.0002 |
| Negative vs. Tween 80 | 0.9999 | 0.1397 | 0.9416 | <0.0001 |
| **Other compounds** |  |  |  |  |
| **Comparison** | **0 hr** | **5 hr** | **10 hr** | **24 hr** |
| Negative vs. 3-Hydroxy 2-Butanone | >0.9999 | 0.9994 | 0.9886 | 0.9997 |
| Negative vs. Group C | 0.9994 | 0.1229 | 0.9089 | 0.9816 |
| Negative vs. Choline | 0.9999 | 0.347 | 0.7008 | 0.9997 |
| Negative vs. Chondroitin Sulfate C | 0.9995 | 0.6108 | 0.9996 | 0.6006 |
| Negative vs. Deferoxamine Mesylate | >0.9999 | 0.0013 | 0.0093 | 0.999 |
| Negative vs. L-Homoserine Lactone | 0.9999 | 0.0037 | 0.0046 | 0.9994 |
| Negative vs. Pyrrolo-quinoline quinone | 0.9999 | 0.626 | 0.9955 | 0.3648 |
| Negative vs. Acetamide | 0.9995 | 0.999 | 0.9996 | 0.9997 |
| Negative vs. Dihydroxy-Acetone | >0.9999 | 0.9997 | 0.9991 | 0.5459 |
| Negative vs. D-Ribono-1,4-Lactone | >0.9999 | 0.1733 | 0.9563 | 0.9187 |
| Negative vs. Glycerol | >0.9999 | 0.0231 | 0.5796 | 0.9996 |
| Negative vs. Gelatin | 0.9993 | 0.7475 | 0.0254 | 0.0038 |
| Negative vs. Gluthathione | 0.9998 | 0.1902 | 0.4263 | 0.9994 |
| Negative vs. Hematin | 0.9999 | 0.0016 | 0.0029 | 0.9991 |
